# Acetazolamide-Assisted Weaning From Mechanical Ventilation in a Child With Mixed Sleep Apnea: A Case Report

**DOI:** 10.7759/cureus.98508

**Published:** 2025-12-05

**Authors:** Saroj Kumar Tripathy, Sarbari Sarkar, Medhagopal R. G., Pradosh Kumar Sarangi, Archana Malik, Sarthak Das

**Affiliations:** 1 Pediatrics, All India Institute of Medical Sciences, Deoghar, Deoghar, IND; 2 Radiodiagnosis, All India Institute of Medical Sciences, Deoghar, Deoghar, IND; 3 Pulmonary Medicine, All India Institute of Medical Sciences, Deoghar, Deoghar, IND

**Keywords:** acetazolamide, adolescent, case report, mixed sleep apnea, obesity

## Abstract

An elevated central apnea-hypopnea index is typically considered indicative of abnormal breathing in children. Treatment of central sleep apnea (CSA) is complex, and multiple treatment options are tried with variable outcomes. Acetazolamide, a carbonic anhydrase inhibitor, is known to induce metabolic acidosis and can stimulate the respiratory drive. We report a 13-year-old boy who was having obesity and mixed apnea with an apnea-hypopnea index of 67.4/hour, who was hospitalized with hypercapnic respiratory failure. He was successfully weaned from prolonged mechanical ventilation following acetazolamide therapy. Post-discharge, he has had no apnea and continues to do well, with an improved quality of life at two months of follow-up. Acetazolamide use for central apnea/mixed apnea in children is very rare and not well established. Our case highlights the potential use of acetazolamide as an adjunct therapy in children with CSA, which needs further exploration.

## Introduction

The American Academy of Sleep Medicine defines central sleep apnea (CSA) as the cessation of airflow, accompanied by absent chest wall and/or abdominal movements, lasting longer than 20 seconds or more than two baseline respiratory cycles, if associated with arousal, awakening, or oxygen desaturation of at least 3% [1]. A central apnea-hypopnea index (CAHI) of five events per hour or more is considered pathological in children [2]. Several therapeutic options have been considered for the treatment of CSA, including supplemental oxygen therapy, bilevel positive airway pressure therapy, and methylxanthines. Acetazolamide, a carbonic anhydrase inhibitor, is known to induce metabolic acidosis and can stimulate the respiratory drive. Weaning from mechanical ventilation in children with central apnea is complex and gradual, with frequent failures and guarded results. Here, we report a case of a 13-year-old boy with difficulty weaning from mechanical ventilation who had a positive outcome following treatment with acetazolamide.

## Case presentation

A 13-year-old male patient was admitted with complaints of frequent sleepiness during the day, poor sleep at night, and frequent awakenings at night for two years. The episodes were frequent and increasing over the last two months. On examination, the child was obese (BMI 23.8) and had stable vital signs: pulse rate (PR) 90/min, respiratory rate (RR) 15/min, blood pressure (BP) 94/60 mm Hg, and SpO_2_ 94% on room air. Systemic examination was unremarkable. The patient underwent polysomnography, which showed an apnea-hypopnea index (AHI) of 67.4/hour, with a central apnea index of 3.4 and an obstructive apnea index of 2.6, and the lowest SpO_2_ of 67%. Although the child had a short neck with obesity, any significant local cause of obstruction needing surgical intervention was ruled out after multispecialty consultation. A diagnosis of mixed apnea was made, and the patient was planned to be put on continuous positive airway pressure (CPAP) therapy. The child was admitted for titration and education regarding CPAP therapy.

On the second day of hospitalization, the child developed hypercapnic respiratory failure (arterial blood gas (ABG): pH 6.84, partial pressure of oxygen (PO_2_) 117 mmHg, partial pressure of carbon dioxide (PCO_2_) 227.2 mmHg, and bicarbonate (HCO_3_⁻) 46 mEq/L) and required endotracheal intubation with mechanical ventilation. Serum electrolyte levels and hemogram reports were normal. On the third day of intubation, the child’s symptoms improved, and an extubation trial was attempted, but without success in view of a rapid rise in PCO_2_ and hypoventilation. He developed a ventilator-associated pneumonia on day 9 of hospitalization, which was appropriately treated with antimicrobials. Given the prolonged mechanical ventilation requirement, a tracheostomy was performed on day 16 of hospitalization, and mechanical ventilation continued via the tracheostomy tube. Once the lung pathology subsided, again the trial of weaning off from the ventilator was started, and cycling between oxygen via T-piece ventilation and mechanical ventilation was initiated, after which the child was able to sustain for only two to three hours a day. Given the patient’s intolerance and complex clinical presentation, alternative therapeutic options were explored.

On day 32 of hospital admission, a trial of oral acetazolamide was initiated to enhance ventilatory drive and prevent apnea. Acetazolamide was started at 6 mg/kg/day, a single dose given before bedtime. Laboratory investigations, including creatinine, sodium, potassium, chloride, and ABG, were monitored during hospitalization. Improvement in RR, oxygen saturation, and tolerance to the T-piece trial increased substantially over the next week. Maximum saturation drop was documented till 89% during sleep. The child remained entirely on room air (PR 70/min, RR 20/min, BP 100/66 mm Hg, and SpO_2_ 97%) without ventilatory support after 10 days of acetazolamide treatment. The child was discharged with a tracheostomy tube in situ and oral acetazolamide therapy. On the two-month follow-up visit, parents reported improved sleep quality, no apnea, and overall health-related quality of life.

## Discussion

Sleep apnea is an adverse outcome of respiratory airflow pauses during sleep, which can be classified into three categories: obstructive sleep apnea (OSA), mixed sleep apnea (MSA), and CSA. MSA can be defined by the presence of both CSA and OSA, due to ventilatory control dysfunction and upper respiratory tract collapsibility. CSA, in the form of abnormal breathing pattern during wakefulness, has been observed in 1-5% of healthy children [3]. Causes of apnea include hypothyroidism, obesity, gastroesophageal reflux, anatomical abnormalities of the brain and brainstem (such as Arnold-Chiari malformation or foramen magnum stenosis), and syndromic conditions such as Prader-Willi syndrome. Upper airway abnormalities can aggravate the CSA component of MSA. A key feature of central hypoventilation syndrome is a lack of increase in ventilation rate in response to hypercarbia or hypoxia. Physiological dysregulation may involve multiple organ systems such as respiratory, cardiac, vasomotor, sudomotor, and neurologic systems. There is no definite management of CSA, and several options like bilevel positive airway pressure, methylxanthines, and acetazolamide have been tried with some success [4]. Acetazolamide, a carbonic anhydrase inhibitor, has been tried in treating patients with CSA through its ability to cause metabolic acidosis and stimulate respiratory drive, lowering the apneic PaCO_2_ threshold. Chemosensitivity or "controller gain" describes the change in ventilation in response to blood gas changes, which promote both central and peripheral chemoreceptors. This mechanism reduces the plant gain (lung's effort to maintain a stable PaCO_2_ level around 40 mmHg) and reduces excessive loop gain values that destabilize breathing [5].

There are multiple studies on acetazolamide therapy with obesity, chronic pulmonary obstructive disease, and CSA in adults. Recent meta-analyses have indicated acetazolamide's potential short-term benefits in treating both OSA and CSA, with a larger effect on CSA in adults. Although the differences in responsiveness in different subtypes of sleep apnea did not reach statistical significance, acetazolamide's effect on AHI in the case of OSA and/or CSA was demonstrated to be dose dependent by reaching a plateau at approximately a 500 mg/day dose. Doses more than this seemed to increase side effects, which adversely affect tolerance and adherence [6].

The role of acetazolamide in CSA or MSA in children is not clear, and only a few case reports are available in the literature. Given the rarity of this condition in children and potential association with genetic or syndromic conditions, the pharmacotherapy is not well studied. Patients with a diagnosis of CSA, Pitt-Hopkins syndrome, and global developmental delay with intellectual disability, who had frequent apneic episodes, were tried with acetazolamide. A consistent dose of 250 mg/day was given to all patients. All four case reports reported significant improvement in apnea hypopnea episodes and oxygen saturation. No adverse events were observed. Similarly, our patient had positive outcomes following acetazolamide treatment. Our case was unique because he was able to be weaned from prolonged mechanical ventilation within 10 days and was apnea-free for two months post-discharge with treatment. A summary of the previously published case reports is presented in Table 1.

**Table 1:** Summary of previously reported pediatric apnea cases treated with acetazolamide CSA: central sleep apnea; CAHI: central apnea-hypopnea index; SpO_2_: oxygen saturation; Y: years; M: male

Author, year	Age/sex	Diagnosis	Treatment	Follow-up duration	Outcome
Sirianansopa and Amin, 2024 [4]	12 Y/M	Acetazolamide as a therapeutic alternative in CSA in a patient with the FBXO28 mutation	Acetazolamide	-	Significant reduction in CAHI, periodic breathing, and nadir SpO_2_.
Verhulst et al., 2012 [7]	(1) 9 Y/M; (2) 21 Y/M	(1) Pitt-Hopkins syndrome; (2) Pitt-Hopkins syndrome	(1) Acetazolamide 250 mg/day; (2) Acetazolamide 250 mg/day	(1) Five months; (2) Four weeks	(1) Apnea frequency reduced, and there was a marked improvement in oxygen saturation. When acetazolamide was stopped after five months, symptoms reappeared, prompting its reintroduction; (2) The most marked effect was on oxygen saturation. Central apnea persisted, although frequency was decreased.
Gaffney and McNally, 2015 [8]	15 Y/M	Intellectual disability, central hyperventilation, and apneic episodes	Acetazolamide 250 mg/day	Two years	Marked reduction of symptoms (frequency and severity of apnea).
Our case	13 Y/M	Obesity, mixed apnea	Acetazolamide 250 mg/day	Two months	No apnea episodes for the last two months. Marked improvement in sleep quality observed.

## Conclusions

This case highlights the promising role of acetazolamide therapy in facilitating ventilator weaning in pediatric patients with MSA and hypercapnic respiratory failure. Limitation of our study includes a single case report with a short follow-up. Although results for the potential use of acetazolamide as primary therapy are not encouraging, it can be used as an adjunctive therapy in complex cases of CSA, when other treatment options do not yield any improvement. Further research involving children is essential to establish the actual outcome and long-term effect.
